# Semiclassical Molecular Dynamics for Condensed Phase NMR Spectroscopy

**DOI:** 10.1021/acs.jctc.6c01206

**Published:** 2026-09-11

**Authors:** Hampus Karlsson, Marco Cazzaniga, Michele Ceotto

**Affiliations:** Dipartimento di Chimica, 9304 Università degli Studi di Milano, via Golgi 19, Milano 20133, Italy

## Abstract

We use the Meyer–Miller formalism and the Caldeira–Leggett model with its associated and discretized spectral density formalism, to explore the impact of harmonic oscillator baths on the spectral line shapes of single- and two-spin I=12 NMR spin systems. The complex coefficients associated with the spin degrees of freedom are treated as classical position, *q*_*n*_, and momentum, *p*_*n*_, variables to investigate the coupling to the classical harmonic bath oscillators. The expressions derived for the motion of the density matrix elements associated with the observable NMR signal and modulated by the bath oscillators are used in the numerical simulations of NMR spectra. Unlike existing relaxation theories such as the Redfield theory, the method is not limited to short, bath correlation times. This is considered a strength of the method, and simulations have been performed at bath correlation times considered to be on the limit or beyond regimes where Redfield theory usually is applicable. Simulated line shapes are considerably broader than those predicted by Redfield theory. These results are discussed in terms of inclusion of nonsecular bath frequencies and how properties of the bath oscillators are defined.

## Graphical abstract

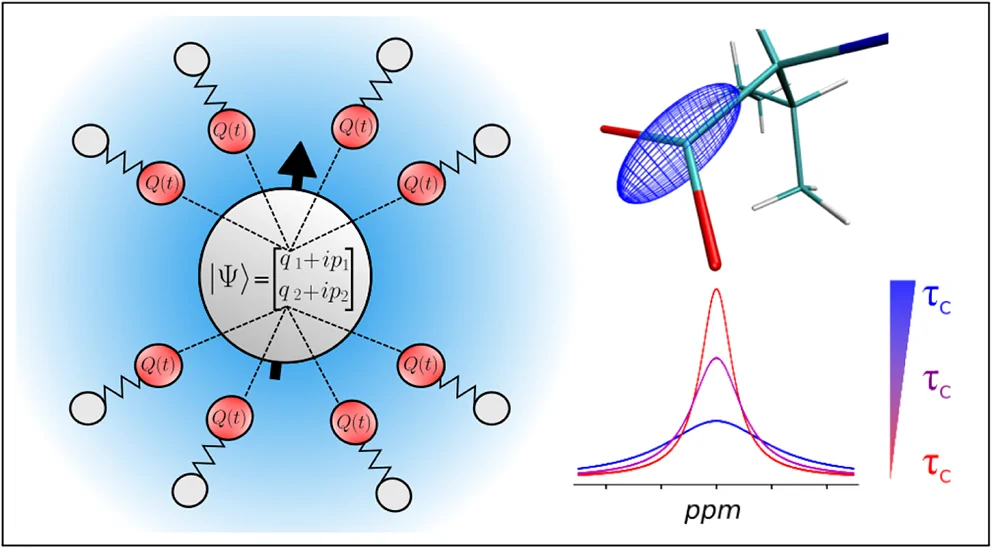

## 1 Introduction

The description of phenomena contributing to the signal line width and longitudinal relaxation within the field of NMR spectroscopy is a challenging topic. In solution-state NMR, when the systems under study are characterized by fast molecular tumbling and short correlation times, Redfield theory^1^ typically gives a comprehensive description of the relaxation and phenomena underlying it. This has led to the fact that Redfield theory is standard NMR textbook material^2–4^ and that nowadays Redfield theory may be used to extract spin relaxation data from the classical molecular dynamics (MD) simulation trajectories.^5^

In solid-state NMR, typically in the presence of magic angle spinning^6^ (MAS), the situation is more complicated when additional, stronger, and anisotropic interactions play a much larger role and affect the relaxation.^7,8^ This is also the case for NMR methods, where the system under study is hyperpolarized with methods such as dynamic nuclear polarization^9,10^ (DNP) and when paramagnetic centers^11^ contribute to the relaxation.

Despite the challenges to describe NMR spin relaxation in such complicated systems, progress has been made in recent years both for hyperpolarized systems^12^ and paramagnetic^13^ systems mainly by reviving the usage of quantum theories, such as the ones by Lindblad.^14^ Within the context of electron spin relaxation and dynamics, other quantum master equations, such as the Nakajima–Zwanzig equation,^15^ have recently been studied. In addition, alternative semiclassical methods such as modified versions^16^ of Schulten and Wolynes’^17^ theory have been proposed for electron spin dynamics. Even if much progress has been made in describing spin relaxation and properties of the NMR spectra for complicated molecular systems, alternative methods are still highly useful.

Thus, in this work, we explore how the semiclassical methods of Meyer & Miller,^18^ in conjunction with the Caldeira–Leggett model^19,20^ for system bath couplings, can be used to describe relaxation effects in different NMR situations. The work by Meyer & Miller,^18^ deeply connects to earlier observations in quantum mechanics,^21,22^ showed that the complex coefficients associated with wave functions and spin states can be interpreted as classical variables. And the Caldeira–Leggett model, originally published in the 1980’s and applied to the problems of quantum Brownian motion^19^ and quantum tunneling,^20^ offers a framework for describing the system bath coupling via the discretized version of the bath spectral density. The model has commonly been used to describe open quantum systems,^23,24^ especially within a path integral^25^ formulation, and due to its connection to phenomena such as tunneling^20^ it was also employed to study the impact of surrounding environments on two-state energy level systems, often referred to as the spin-boson problem.^26–28^ The spin-boson problem, as typically encountered in standard literature,^26,27^ uses the formalism associated with the two energy levels of a spin I=12 particle to model other physical phenomena associated with discrete energy levels. Such phenomena could, for instance, be electronic transitions in molecular systems.^29–35^ The high similarity of the spin-boson problem and a spin in the NMR situation has been pointed out by Leggett *et al.* ^26^ and there are recent examples in the literature where the formalism has been explored for NMR purposes.^36^

In line with ref 36 and in the following sections, we will outline the theory for how the Caldeira–Leggett model and the spin-boson formalism may be adapted for numerical simulations of line broadening and relaxation phenomena in NMR. Since the method relies on numerical evolution of the spin and bath oscillators, the method is not limited to certain correlation time regimes, which is considered a strength of the method. We also choose to work within the theoretical framework pointed out by Meyer and Miller,^18^ Strocchi,^22^ and Dirac^21^ and work with the spin degree of freedom in terms of classical position and momentum variables. This approach allows one to intuitively rationalize the coupling between the spin degree of freedom and environmental variables. It also paves the way for future work, since the representation of the spin degree of freedom in terms of position and momentum variables would open up for adapting methods such as the semiclassical initial value representation^37^ (SC-IVR) also for NMR spin dynamical problems.

## 2 Theory

### 2.1 Classical Representation of the Spin Bath Hamiltonian

The Caldeira–Leggett model^19,20^ for the spin-boson problem^26,27^ models a quantum system coupled to a set of Harmonic oscillators in a surrounding environment or a ″bath″. The oscillations of the environment will drive transitions of the quantum system. The Hamiltonian for the spin-boson problem as it is typically encountered in literature, is

1 $$Ĥ={Ĥ}_{S}+{Ĥ}_{B}+{Ĥ}_{SB}$$

where *Ĥ*_*S*_, *Ĥ*_*B*_ and *Ĥ*_*SB*_ represent separate Hamiltonians for the system, the bath and the system bath coupling. They are defined as

2a $${Ĥ}_{S}=\left[\begin{array}{ll} 0 & \Delta \\ \Delta & 0 \end{array}\right]$$

2b $${Ĥ}_{B}=\underset{j}{\sum }\left[\begin{array}{ll} \frac{1}{2}\left(P_{j}^{2}+\omega_{j}^{2}Q_{j}^{2}\right) & 0\\ 0 & \frac{1}{2}\left(P_{j}^{2}+\omega_{j}^{2}Q_{j}^{2}\right) \end{array}\right]$$

2c $${Ĥ}_{SB}=\underset{j}{\sum }\left[\begin{array}{ll} -C_{j}Q_{j} & 0\\ 0 & C_{j}Q_{j} \end{array}\right]$$

where *Q*_*j*_ and *P*_*j*_ represents position and momentum variables for the *j* ^*th*^ oscillator of the bath. *ω*_*j*_ and *C*_*j*_ represent the oscillation frequency and coupling to the same oscillator. Δ represents a ″coupling″ or ″tunneling splitting″ term and it is this term that will drive transitions between the two different states. As can be understood from studying the different terms in eq 2, during time-evolution of the system, transitions between the two different spin states will occur when the *Ĥ*_*SB*_ term takes on small or zero values relative to the Δ term. This is completely analogous to ″on resonance″ radio frequency irradiation in NMR. We will now proceed, to adapt the Hamiltonian in eqs 1 and 2 for the NMR situation. Our primary interest is not to study processes that induce transitions between the two states (longitudinal relaxation or radio frequency irradiation). Instead, we wish to study line-broadening phenomena or dephasing of an ensemble of spins. To do this we will replace the system Hamiltonian in eq 2 with the Zeemann or chemical shift Hamiltonian for a single spin I=12 :

3 $${Ĥ}_{z}=\Omega {Î}_{z}$$

We also let the oscillators couple to *î*_*z*_ and introduce the factor 12 for the coupling term. We then remove the hats from the *Ĥ*_*B*_ and *Ĥ*_*SB*_ symbols and let them represent their individual sums in *Q*_*j*_ and *P*_*j*_, i.e., from now on they have the classical meaning, $H_{B}=\underset{j}{\sum }\frac{1}{2}\left(P_{j}^{2}+\omega_{j}^{2}Q_{j}^{2}\right)$ and *H*_*SB*_ = ∑_*j*_ *C*_*j*_ *Q*_*j*_. The Hamiltonian obtained by these modifications is diagonal and has the following appearance:

4 $$Ĥ=\left[\begin{array}{ll} H_{B}+\frac{\Omega }{2}-\frac{H_{SB}}{2} & 0\\ 0 & H_{B}-\frac{\Omega }{2}+\frac{H_{SB}}{2} \end{array}\right]$$

Once the quantum mechanical matrix Hamiltonian is defined, we may proceed in the footsteps of Meyer & Miller^18^ and others,^22^ and convert the quantum mechanical matrix Hamiltonian into a ″classical″ Hamiltonian function. This is done by defining the real and complex parts of the complex coefficients (*c*_*n*_) associated with the Zeemann eigenstate vector as position and momentum variables:

5 $$q_{n}=\frac{2Re\left(c_{n}\right)}{\sqrt{2}},,p_{n}=\frac{2Im\left(c_{n}\right)}{\sqrt{2}}$$

Notice that we use lower script *n* for indexing variables associated with the spin degree of freedom, likewise we used *j* to index variables associated with different bath oscillators. We can now proceed and write the Zeemann eigenstate as

6 $$\left|\Psi \right\rangle =\frac{\sqrt{2}}{2}\left[\begin{array}{l} q_{1}+ip_{1}\\ q_{2}+ip_{2} \end{array}\right]$$

We obtain the ″classical″ Hamiltonian function (*H*_*CL*_) by using the expression for the expectation value, *H*_*CL*_ = ⟨Ψ|*Ĥ*|Ψ⟩, and the definition of the eigenstate, |Ψ⟩ in eq 8, the matrix Hamiltonian in eq 6 yields the following ″classical″ Hamiltonian function:

7 HCL=Ω4(p12−p22+q12−q22)+HB2(p12+p22+q12+q22)+HSB4(q22+p22−q12−p12)

and then we obtain from *H*_*CL*_ the equations of motion for the *q*_*n*_ and the *p*_*n*_ variables by calculating:

8 $${\mathrm{q̇}}_{n}=\frac{\partial H_{CL}}{\partial p_{n}},,{ṗ}_{n}=-\frac{\partial H_{CL}}{\partial q_{n}}$$

and obtaining the following set of equations of motion:

9a $${\mathrm{q̇}}_{1}=H_{B}p_{1}-\frac{H_{SB}}{2}p_{1}+\frac{\Omega }{2}p_{1}$$

9b $${\mathrm{q̇}}_{2}=H_{B}p_{2}+\frac{H_{SB}}{2}p_{2}-\frac{\Omega }{2}p_{2}$$

9c $${ṗ}_{1}=-H_{B}q_{1}+\frac{H_{SB}}{2}q_{1}-\frac{\Omega }{2}q_{1}$$

9d $${ṗ}_{2}=-H_{B}q_{2}-\frac{H_{SB}}{2}q_{2}+\frac{\Omega }{2}q_{2}$$

Wang *et al.* ^32^ points out that a drawback of this set of equations is that the bath term (*H*_*B*_) becomes very large in magnitude when the bath contains many oscillators and span a large frequency range, rendering the set of equations a stiff system, making numerical integration a formidable task. Their suggestion to alleviate this problem is to do the following variable substitutions:

10a $$q_{1}^{\prime }=\left(q_{1}^{2}+p_{1}^{2}+q_{2}^{2}+p_{2}^{2}\right)$$

10b $$p_{1}^{\prime }=\left(q_{1}^{2}+p_{1}^{2}-q_{2}^{2}-p_{2}^{2}\right)$$

10c $$q_{2}^{\prime }=\left(p_{2}q_{1}-p_{1}q_{2}\right)$$

10d $$p_{2}^{\prime }=\left(q_{1}q_{2}+p_{1}p_{2}\right)$$

The equations of motion for these substitution variables are

11a $$\frac{d}{dt}\left(q_{1}^{2}+p_{1}^{2}+q_{2}^{2}+p_{2}^{2}\right)=0$$

11b $$\frac{d}{dt}\left(q_{1}^{2}+p_{1}^{2}-q_{2}^{2}-p_{2}^{2}\right)=0$$

11c ddt(p2⁡q1−p1⁡q2)=Ω(q1⁡q2+p1⁡p2)−HSB(q1⁡q2+p1⁡p2)

11d ddt(q1⁡q2+p1⁡p2)=−Ω(p2⁡q1−p1⁡q2)+HSB(p2⁡q1−p1⁡q2)

Details of their derivation may be found in the Supporting Information. As discussed by Wang *et al.*,^32^ the point of the variable substitutions in eqs 10a–d, is that it gets rid of the problematic *H*_*B*_ term and thus solves the stiffness problem. It is also worth pointing out that the derivatives in eqs 19 and 20 evaluate to zero. This is only the case when there are no off-diagonal terms in the original matrix Hamiltonian, which would translate to absence of any radio frequency terms in the NMR context. We now proceed and use the Zeemann eigenstate earlier defined in eq 8, and calculate the density matrix formed by the outer product of |Ψ⟩, but we represent it using the *q*_*n*_/*p*_*n*_ variables defined above:

|Ψ⟩⟨Ψ|=12[q12+p12p1⁡p2+ip1⁡q2−ip2⁡q1+q1⁡q2p1⁡p2−ip1⁡q2+ip2⁡q1+q1⁡q2q12+p12] 12

Now we can appreciate that the integration variables of eqs 17 and 18 corresponds to the real and imaginary parts of the off-diagonal elements of the density matrix, which in turn represents the +/- coherences that we want to detect in an NMR experiment. Thus, the problem of simulating a single spin NMR spectrum, taking into account broadening from a set of harmonic oscillators in a surrounding environment using the Caldeira–Leggett model boils down to solving eqs 21 and 22. Now, considering the direct correspondence between the integration variables in terms of *q*_*n*_/*p*_*n*_ and the density matrix elements and if we let *ρ*_21_ represent the lower diagonal element of the density matrix, the solution to eqs 21 and 22 may also be written:

13 $$\rho_{21}\left(t\right)=\rho_{21}\left(0\right)e^{i(\Omega -H_{SB})t}$$

which is further explained in the Supporting Information. *H*_*SB*_ in eq 24 will be time-dependent due to the evolution of the bath oscillators and a step-wise solution of eq 24 will be necessary. In the scenario we consider here we assume that the Ω is time-independent, such as would be the case of isotropic chemical shift only. However, in the field of NMR there might be many situations where also Ω has a complicated time-dependence.

### 2.2 The Bath Oscillators and Discretized Spectral Density

We have so far not addressed properties of the bath and the equations of motion for the bath oscillators, which are obtained by replacing *H*_*B*_ and *H*_*SB*_ in eq 6 with their explicit expressions for the bath variables. We then repeat the steps in eqs 8–10 but calculating the partial derivatives in eq 10 with respect to *Q*_*j*_ and *P*_*j*_, as described in the Supporting Information. This gives the equations of motion for the *j* ^*th*^ oscillator:

14a $${\mathrm{Q̇}}_{j}=P_{j}\left(q_{1}^{2}+p_{1}^{2}+q_{2}^{2}+p_{2}^{2}\right)$$

14b Ṗj=−Qj⁡ωj2(q12+p12+q22+p22)+Cj2(q12+p12−q22−p22)

Equations 25 and 26 once again remind us of the meaning of the substitution variables in eqs 10a–d. The term, $\left(q_{1}^{2}+p_{1}^{2}+q_{2}^{2}+p_{2}^{2}\right)$ occurring in eqs 25 and 26 corresponds to the sum of the diagonal elements of the density matrix,^32^ which remains constant (as also implied by eq 19). For the $\left(q_{1}^{2}+p_{1}^{2}-q_{2}^{2}-p_{2}^{2}\right)$ term, representing the difference in population between the two states, the situation is different. This term might change if there were any mechanisms, to drive such a change, which in the NMR context would be for instance the presence of a radio frequency fields or mechanism for longitudinal relaxation which we don’t consider in this case. This term also highlights an important aspect of the Caldeira–Leggett model, which is that the change in the spin state/state of the two-level system will affect the bath oscillators, i.e., not only does the environment affect the two-level system, but also the two-level system affect the environment.^26^ In the work considered here, i.e., where we aim to simulate NMR spectra, it is the ″precession of the magnetization vector in the x,y-plane″ or more formally, the oscillation of *ρ*_21_(*t*), stemming from the solution of eq 24 above, that we are interested in. In the NMR experiment, the coherence oscillation that we want to ″record″ and calculate, is created when the spin system is prepared in a superposition state between the high and low energy states, and to give maximum signal the system is prepared such that both states are equally populated. This means that for simulating the transverse magnetization associated with recording the free induction decay (FID) resulting in the NMR spectrum after Fourier transform, both states are typically equally populated and the $\left(q_{1}^{2}+p_{1}^{2}-q_{2}^{2}-p_{2}^{2}\right)$ term in eq 26 becomes equal to zero. The fact that this term remains zero in our case is a major difference compared to the conventional formulation of the spin-boson formalism. More specifically, when we use the harmonic oscillators to simulate the dephasing of the spin ensemble in the absence of any diagonal terms in the original Hamiltonian, there will be no feedback from the spin-system on the surrounding bath. So, with the difference term in eq 26 equal to zero, and with the insight that the $\left(q_{1}^{2}+p_{1}^{2}+q_{2}^{2}+p_{2}^{2}\right)$ term remains constant, we can get away with treating the bath oscillators as individual oscillators and we may thus use the analytical solution:

15 $$Q_{j}\left(t\right)=Q_{j}\left(0\right)\cos\left(\omega_{j}t\right)+\frac{1}{\omega_{j}}P_{j}\left(0\right)\sin\left(\omega_{j}t\right)$$

for the time-evolution of the position of the *j*th bath oscillator. To complete the description of the model of the bath, we also need to define the spectral density. To get a physical basis for this we choose to proceed with a density

16 $$J\left(\omega \right)=\frac{\tau_{c}}{1+\omega^{2}\tau_{c}^{2}}$$

which is the spectral density familiar to NMR spectroscopists from Redfield theory for the description of relaxation induced by mechanisms such as chemical shift anisotropy (CSA) and the dipolar coupling,^3^ and where *τ*_*c*_ represents the bath correlation time. This is also the logical choice for us, since we let the *H*_*SB*_ term couple to *î*_*z*_ in the matrix Hamiltonian of eq 6, implying a modulation of the chemical shift. The spectral density in eq 28 is in turn scaled by two different factors to include the effects of relaxation caused by CSA.^3^ The first scaling factor is $\xi_{CSA}^{2}$ defined as

17 $$\xi_{CSA}^{2}=\frac{1}{15}{\left(\gamma_{13C}B_{0}\right)}^{2}{\left(\Delta \sigma \right)}^{2}$$

where *γ*_13*C*_ is the gyromagnetic ratio for a ^13^C nucleus, *B*_0_ is the external magnetic field and Δ*σ* is the chemical shift anisotropy^3,7,11,38^ which is defined as

18 $$\Delta \sigma =\sigma_{zz}-\frac{1}{2}\left(\sigma_{xx}+\sigma_{yy}\right)$$

which is the definition according to Kowalewski.^3^ In eq 30, *σ*_*xx*_, *σ*_*yy*_ and *σ*_*zz*_ represent the chemical shielding anisotropy tensor components in the principal axis system (PAS). The second scaling factor we used to scale the spectral density in eq 28 is (1 + *η* ^2^/3) and it accounts for the asymmetry, *η* of the chemical shielding tensor,^3,7,11,38,39^ and it is defined as

19 $$\eta =\frac{3\left(\sigma_{xx}-\sigma_{yy}\right)}{2\Delta \sigma }$$

For the discretization of the spectral density according to the ideas of Caldeira and Leggett,^19,20,26^ we use the definition of the discretized spectral density as

20 $$J\left(\omega \right)=\frac{\pi }{2}\underset{j}{\sum }C_{j}^{2}\delta \left(\omega -\omega_{j}\right)$$

which occur in the work by Stock^29^ and where the coupling coefficients (*C*_*j*_) are interpreted as dimensionless ones, so as for the position (*Q*_*j*_) and momentum (*P*_*j*_) variables of the bath oscillators. How the discretized spectral density in eq 32 and the position and momentum variables of the bath oscillators are defined have important consequences for the simulations, and deeply connects to what physical mechanisms that one assumes play a role in the interaction between the bath and spin. Regarding how to choose discrete frequencies for the bath oscillators we use the sampling scheme^32^ which gives a higher sampling density at lower frequencies.

21 $$\omega_{j}=\frac{j^{2}}{N^{2}}\omega_{max},j=1,...,N$$

where *ω*_*max*_ is the highest frequency occurring in the bath investigated and *N* is the number of discrete oscillators. For calculation of the coupling constants associated with each discrete frequency, the middle points between *ω*_1_, ..., *ω*_*N*_ are used as limits of integration and the continuous spectral density in eq 28 is integrated according to

22 $$\int_{\omega_{j-1/2}}^{\omega_{j+1/2}}\frac{\tau_{c}}{1+\omega^{2}\tau_{c}^{2}}d\omega =arc\;\tan\left(\tau_{c}\omega_{j+1/2}\right)-arc\;\tan\left(\tau_{c}\omega_{j-1/2}\right)$$

where the integration limits, *ω*_*j*+1/2_ = *ω*_*j*_ + (*ω*_*j*+1_ – *ω*_*j*_)/2 and *ω*_*j*–1/2_ = *ω*_*j*_ – (*ω*_*j*_ – *ω*_*j*–1_)/2 are the middle points between the discrete frequencies. The analytical expression for the area *A* under the unscaled spectral density is the right hand side of eq 34. This is then scaled with the CSA constants and the coupling coefficients between the spin and the bath oscillators are calculated as

23 $$C_{j}=\sqrt{\frac{2}{\pi }\xi_{CSA}^{2}\left(1+\eta /3\right)A}$$

such that the summation of *C*_*j*_ associated with the discretized spectral density of eq 32 recreates the scaled, continuous spectral density.

### 2.3 Extension to Coupled Spin Systems

The theories introduced in the two previous sections can easily be extended to multi-spin systems. First, we change the system Hamiltonian *Ĥ*_*S*_ in eqs 1 and 2 to be instead

24 $${Ĥ}_{S}=\Omega_{1}{Î}_{1z}+\Omega_{2}{Î}_{2z}+\pi J2{Î}_{1z}{Î}_{2z}$$

This is a two-spin NMR Hamiltonian where a term for *J*-coupling between the spins is included. In such systems, we may let the system bath coupling terms couple to *î*_1*z*_ and *î*_2_ *_z_,* respectively. Separate spectral densities for the different spins is then defined and employed. Then, by following the steps outlined in Section 2.1 with the extension of *q*_*n*_/*p*_*n*_ variables and vectors for the multi-spin system, we derive equations for the two-spin case as well (see the Supporting Information). Just as in the one-spin case, the goal is to obtain equations of motion for the time-evolution of the density matrix elements associated with the so called ″–1 quantum coherences″ or single-quantum coherence. This is the lower off-diagonal matrix elements that connect diagonal elements differing by −1 in their spin quantum number. Analogously to what have been done for the one-spin derivation, we obtain equations of the type (see the Supporting Information):

25 $$S\left(t\right)=\rho_{31}\left(0\right)e^{i(-H_{SB1}+J\pi +\Omega_{1})t}+\rho_{42}\left(0\right)e^{i(-H_{SB1}-J\pi +\Omega_{1})t}$$

where *S*(*t*) represents the time-evolution of the signal for one spin in the two-spin system. As in the single spin case in eq 24, a step-wise solution of the eq 37 is necessary due to the time-dependency of *H*_*SB*1_.

## 3 Methods

### 3.1 Numerical Simulations

NMR free induction decays (FIDs) where simulated by a step-wise solution of eqs 24 and 37. Simulations were done using an absolute Larmor frequency or lab frame chemical shift of 100 MHz and bath oscillation frequencies within the range 0 < *ω*_*j*_ ≤ 200 MHz. Time steps in all simulations were set to 2.5 × 10^–9^ s, such that Δ*t* = (2*ω*_*max*_)^−1^. Discrete bath frequencies and coupling constants where calculated according to eqs 32–35. The position variables of the bath oscillators, *Q*_*j*_ where evolved according to the analytical solution for the harmonic oscillator in eq 27. Bath oscillators were evolved in time and then scaled by their corresponding coupling constants, *C*_*j*_ prior to summation and inclusion in the solution of eqs 24 and 37. Bath position (*Q*_*j*_) and momentum (*P*_*j*_) variables were treated as unitless variables and randomly sampled from zero-centered normal distributions with a standard deviation of one. 4096 bath oscillators and 2048 spin/bath trajectories where used in the simulations of single spin spectra. In the single spin case the simulated FIDs spanned 10.5 ms (for *τ*_*c*_ = 100 ns and *τ*_*c*_ = 50 ns) and 21 ms in the *τ*_*c*_ = 10 ns case. For the two spin spectrum, 1024 trajectories and 8196 bath oscillators were used and the simulated FID spanned 42 ms. The continuous spectral density in eq 28 was scaled according to the anisotropy and asymmetry (see for example Kowalewski^3^ of a CSA tensor with principal axis frame (PAS) shieldings, *σ*_11_ = −11.95 ppm, *σ*_22_ = 55.24 ppm and *σ*_33_ = −87.02 ppm. The chemical shieldings were calculated for a isoleucine (COD ID: 2003104 ^40^ carbonyl carbon nucleus using Quantum Espresso^41,42^ /GIPAW with *.pbe-rrkjus-gipaw-dc.UPF pseudopotentials,^43^ see Figure 1. Simulations were run either as multi-threaded C++ code on 96 cores of one node of the Data Centric General Purpose (DCGP) partition of the CINECA Leonardo^44^ super computer cluster or in house on a 64 core Silicon Graphics UV2000 system. Simulations were also run as hybrid CUDA/C++ code on a 8GB Jetson Orin Nano single board computer, where the 1024 CUDA GPU cores were used to evolve the bath oscillators. Simulated FIDs were then zero-filled and Fourier transformed into spectra using the fftw3 library.^45^

**Figure 1:**
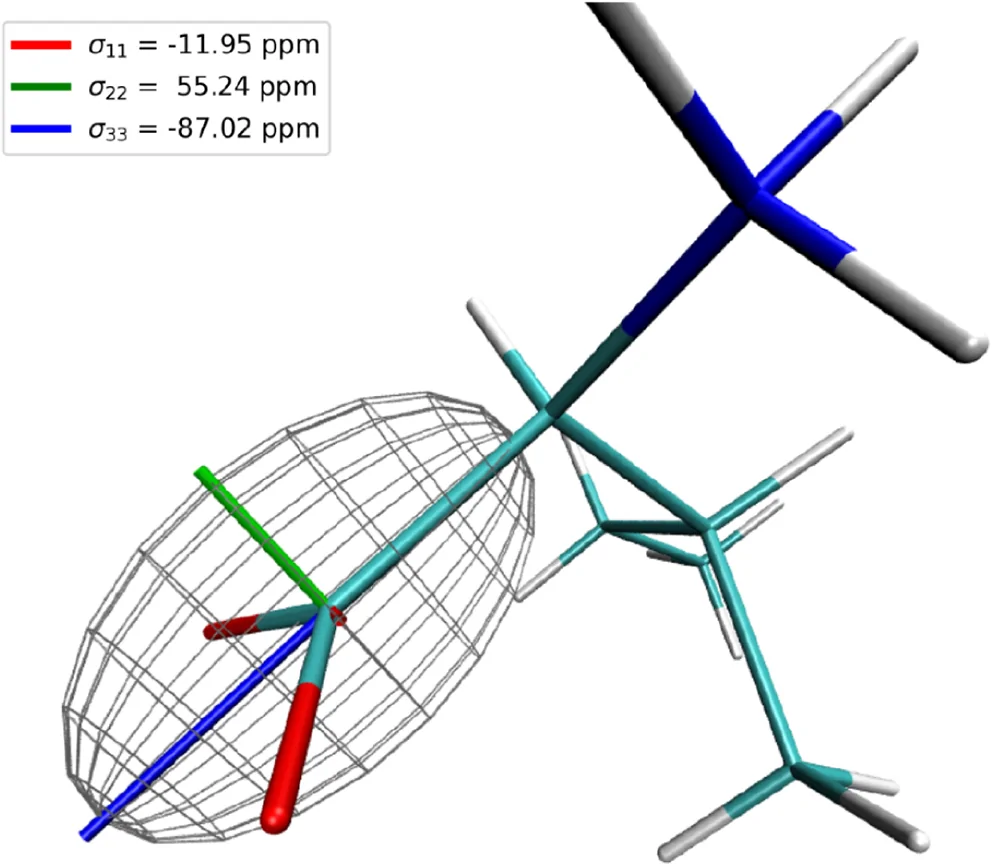
Orientation of the chemical shift anisotropy tensor and its associated principal axis components on the carbonyl carbon of isoleucine. Notice the very small *σ*_11_ component (red) close the carbon atom center.

## 4 Results and Discussion

### 4.1 Bath Properties

To see how well the Caldeira–Leggett, discretized bath could describe line-broadening effects in NMR spectra, we performed simulations according to the theory outlined in Section 2 for a set of different bath correlation times, in the vicinity of the period of the Larmor frequency. In Figure 2, we show properties of the bath and its oscillators. As may be understood from Figure 2a, the sampling of bath modes according to eq 33 causes the low frequency bath modes to be sampled with considerably less spacing. This is then mirrored in Figure 2c, where the corresponding coupling coefficients are of lower magnitudes, despite the higher intensity of the spectral density in the very low frequency range, seen in Figure 2b. The sampling scheme of the continuous spectral density and the frequency range investigated need to be chosen carefully, since they will both affect the efficiency of the numerical calculations and may introduce unwanted artifacts if not thought through. In this work we chose bath correlation times in the range of 10^–7^–10^–8^ *s*, and a Larmor frequency of 100 MHz. These are values that are realistic for a ^13^C nucleus in a very large^46^ protein in a 400 MHz spectrometer. We have also chosen the spectral density to be scaled by the chemical shielding anisotropy of a carbonyl carbon nucleus, which has a high anisotropy (−108.66 ppm). These choices have several consequences. Firstly, the relatively low Larmor frequency in combination with the rapidly decaying spectral density functions for these correlation times and the large anisotropy fo the carbonyl carbon nucleus allows us to limit the step-size in the simulations, because we ignore high frequencies where the spectral density has low intensity. It also highlights an important and advantageous aspect of the method, which is that the simulation relies on a numerical evolution of the spin and bath variables and not on perturbative series expansions that in turn relies on truncations under assumptions of short correlation times, as is the case for Redfield theory.^1,3^ The method is not limited to certain correlation time regimes. Because of this we also included correlation times, such that *τ*_*c*_ ≥ Ω^–1^, which, is a highly interesting parameter range to study.

**Figure 2:**
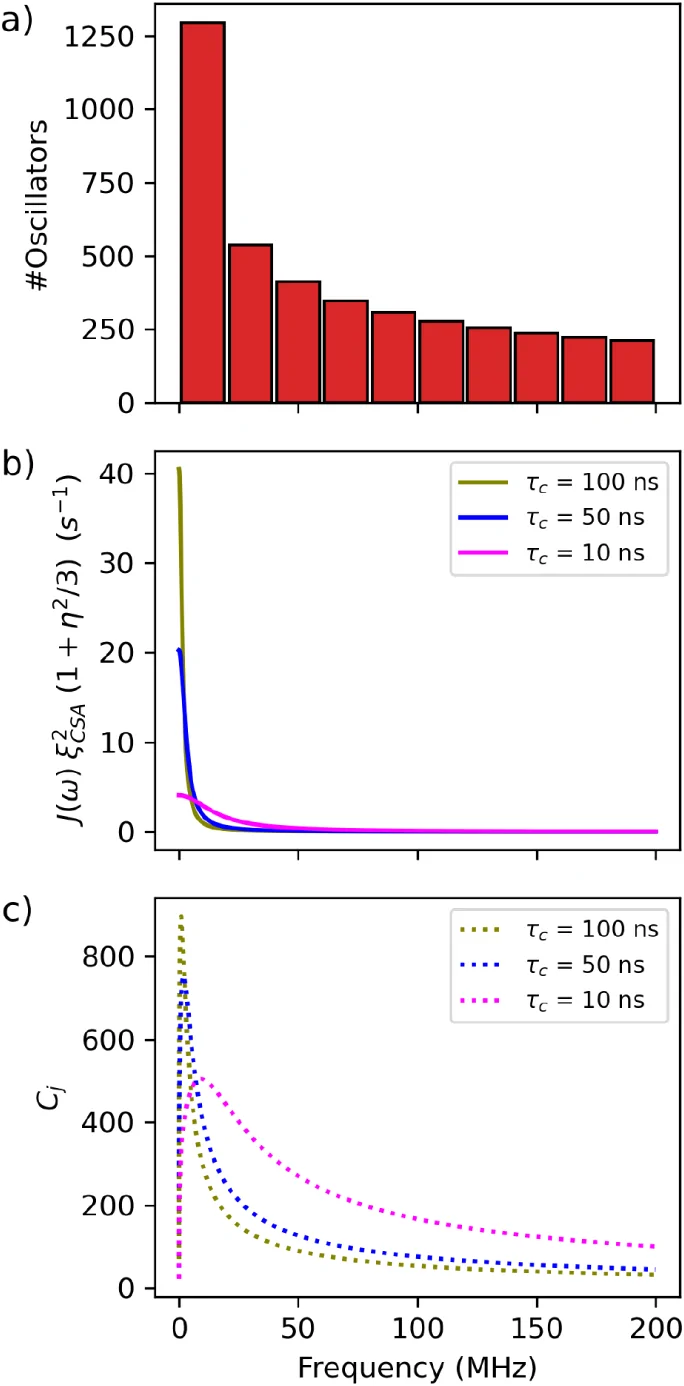
Properties of the bath. a) The number of bath oscillators in different frequency intervals according eq 33 for 4096 oscillators, used in all single spin simulations. b) The spectral densities and associated bath correlation times used for the simulations scaled according to the anisotropy and asymmetry of a CSA tensor with PAS frame shieldings, *σ*_11_ = −11.95 ppm, *σ*_22_ = 55.24 ppm and *σ*_33_ = −87.02 ppm, respectively. c) Magnitude of the coupling coefficients between the spin ″position″ (*q_n_*) variables and bath oscillator position variables (*Q_j_*), according to the Caldeira–Leggett model and eqs 32–35.

Although the method is not limited to certain correlation time regimes, we choose the regimes where the bath correlation times are on par with the evolution of the chemical shift or the density operator element of interest, because these are the most interesting and feasible scenarios to study when the Larmor frequency isn’t too high. Including higher bath or chemical shift frequencies would necessitate a smaller step-size during the step-wise solving of the equations. For isotropic, rotational diffusion of the bath, which is implicitly assumed here by the type of spectral density chosen in eq 28, it is well-known that low molecular mass and increased temperature would shorten the correlation time.^3^ Actually, low molecular masses and higher temperatures would generate a bath correlation time regime where the spectral density has considerable amplitude at higher frequencies, much higher than the chemical shift or density operator evolution considered here, i.e., the bath becomes much faster than the system. This scenario can also be simulated with the methods implemented in this work with sufficiently long calculation times. However, since those regimes are already well-described by the existing, perturbative master equation approaches^1,28^ we can look at the slower bath correlation time regimes and incorporate other features of the Caldeira–Leggett model, such as when the coupling to the transverse spin operators, or other mechanisms, create feedback from the spin to the bath oscillators. Under such circumstances, we can study the interplay between the change in spin state and the low frequency bath oscillators. In this case, the numerical solutions for the evolution of the bath oscillators would be necessary, since the $\frac{C_{j}}{2}\left(q_{1}^{2}+p_{1}^{2}-q_{2}^{2}-p_{2}^{2}\right)$ term in eq 14 ​​​ will no longer be zero and vary with the spin state. Numerical solutions to the bath oscillator equations would also be necessary if more complicated baths will be considered, such as anharmonic baths or if the spin-bath coupling of the coupling Hamiltonian, *H*_*SB*_ in eq 4 will have a quadratic or higher order dependence in *Q*_*j*_. In these cases, numerical methods, such as the Velocity-Verlet algorithm, could be used to evolve the bath oscillators. Although this probably is within our reach, it is a considerably more intense computational challenge, though it is an interesting area for continued efforts. For the scope of this work we take advantage of the assumption of a harmonic bath, perfect superposition of the spin between the Zeemann eigenstates and no mechanisms for changing this spin state, such that we can use the analytical solution for the oscillators in eq 27, which simplifies the computational challenge (see the Supporting Information for additional discussion and data on the simulation times). Regarding possible numerical artifacts, two possible caveats should be considered. Firstly, if a scenario is chosen such that the bath has short correlation time and the spectral density shows considerable amplitude at high frequencies, care has to be taken such that the step-size is sufficiently short to represent the higher frequencies correctly. Secondly, if very low bath frequencies are included in the simulations, they would have incomplete oscillation periods within the total simulation time. In that case, they might act as static additions or subtractions to the chemical shift and may add ″homogeneous″, gaussian contributions to the line shape. Also one should consider that low frequencies do not contribute to a proper dephasing of the ensemble of spins when simulated.

### 4.2 Simulated Line Shapes

In Figure 3, we show the simulated spectra and resulting line shapes in the single spin, i.e., non-coupled case, for the baths and different correlation times investigated. The line shapes agree well with the fitted Lorentzian functions (dashed lines) and the line widths decrease with shorter correlation time. The line widths of 257, 134, and 36 Hz respectively are considerably broader than what would be calculated from Redfield theory^1,3^ which would predict line widths of 54, 27, and 5.5 Hz respectively, once calculated as $\frac{4}{3}J\left(0\right)+J\left(\Omega \right)$ for the correlation times and CSA parameters used.

**Figure 3:**
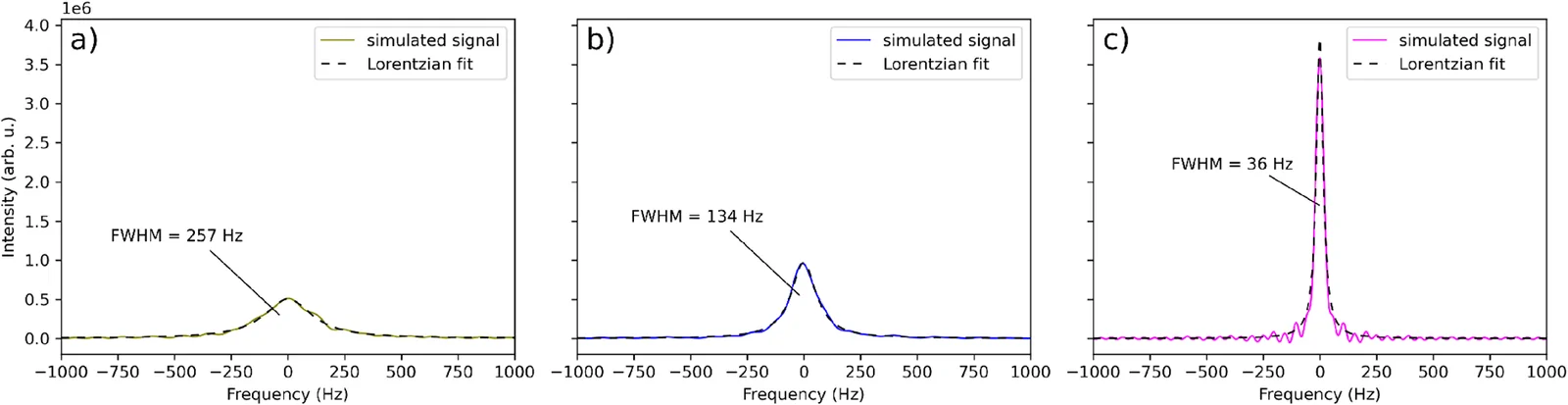
Simulated spectra according to the Caldeira–Leggett model and its associated discretized spectral density for the three different correlation times: a) *τ_c_* = 100 *ns*, b) *τ_c_* = 50 *ns*, c) *τ_c_* = 10 *ns*. The simulations have been done at an absolute Larmor frequency of 100 MHz. The spectra above have been calibrated such that the simulated signals appear at 0 Hz to aid visual inspection. Dashed lines are fitted Lorentzian line shapes and the values in Hz in the spectra are the ″full width at half maximum″ (FWHM) values, from the fitted Lorentzians.

This is a big discrepancy, which is about five times larger line widths from our simulations, with respect to the discretized Caldeira–Leggett bath spectral density compared to Redfield theory. But there are also major differences between the two theories, Redfield theory tells us to only evaluate the spectral density at the zero frequency or at the Larmor frequency due to the secular approximation. Instead, the Caldeira–Leggett model^19,20,26^ aims to recreate the whole area under the spectral density via the use of many discrete frequencies and the coupling constants associated with its discretized form. The other major, important factor affecting the results is how the spin bath coupling constants *C*_*j*_ and oscillator position, *Q*_*j*_, and momentum, *P*_*j*_ variables are defined. We have treated them as dimensionless quantities and sampled *Q*_*j*_ and *P*_*j*_ from zero-centered normal distributions. However, this is a somewhat empirical choice and is not a complete physical description of the bath oscillators and their thermodynamic properties.

### 4.3 Line Widths and Presence of *J*-Coupling

As introduced in Section 2.3, the Caldeira–Leggett model may be also used in the case of coupled spin systems. In the coupled spin case, we allow the bath oscillators to separately couple to both *î*_1*z*_ and *î*_2*z*_ respectively. And just like in the single spin case, simulations can be performed such that the bath oscillators frequency modulate the chemical shift evolution for the individual spins in the ensemble, creating a dephasing and broadening of lines also in the coupled, two spin case. Figure 4, shows the resulting spectrum from the numerical evaluation of eq 37 for the signal of the first spin in a coupled spin pair. On the contrary to the single spin simulations in Section 4.2 the simulation in the coupled spin case in Figure 4 was done using 8196 bath oscillators and the simulated FID time was 42 ms. We tested also shorter FID simulation times. However, we obtained more wiggled spectra to such an extent that the simulated line shapes shifted slightly and not reproduced the *J*-coupling correctly.

**Figure 4:**
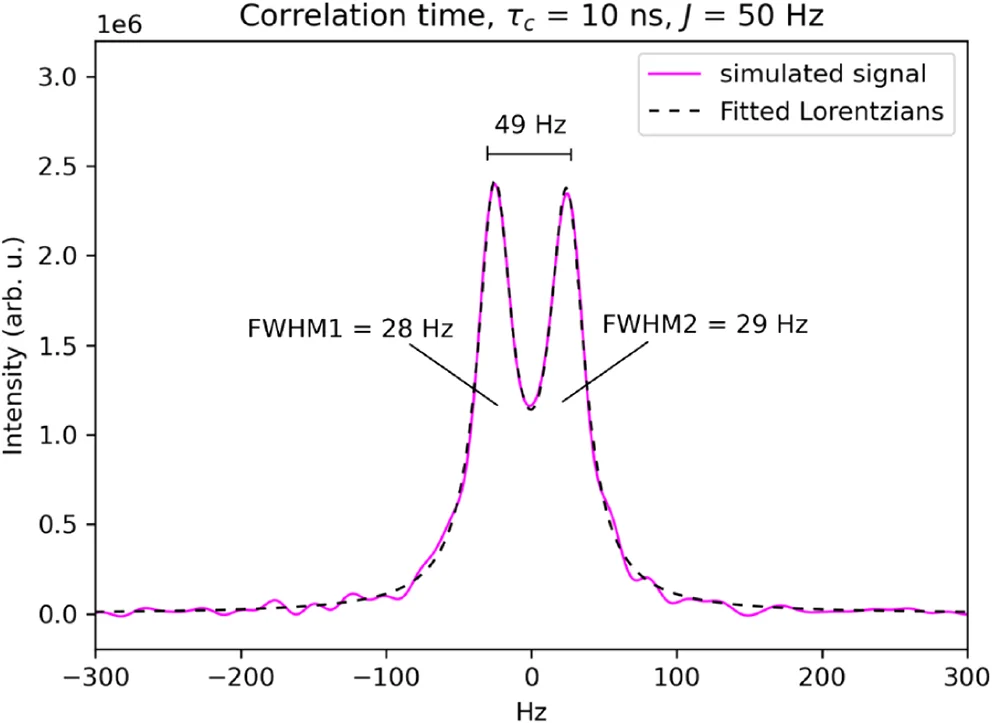
Simulated NMR signal line shape for spin-one in a *J*-coupled spin pair with a coupling constant of 50 Hz and where the spin couples to 8196 bath oscillators. The 49 Hz indicate the distance between the two fitted Lorentzian peaks.

This highlights the importance of including a sufficient number of bath oscillators and simulating long enough time spans in the two spin case, otherwise wiggles may appear in the spectrum because of the abruptly interrupted simulated FID.

In the case of coupled spin systems, the evolution and relaxation effects on density matrix elements other than those covered by eq 37 might also be of high interest to the NMR spectroscopist. We have, however, considered it beyond the scope of this study and the topic of future work to investigate the performance of the Caldeira–Leggett and Meyer and Miller frameworks for the description of these phenomena.

## 5 Conclusions

In this work, we have shown that the ideas of Meyer and Miller in conjunction with the bath discretization methods of Caldeira and Leggett can be modified and adapted for simulating line broadening effects in NMR spectra. The modified formalism is different from its predecessors in the sense that our adaptation concerns the modeling of dephasing of quantum coherences, instead of transitions between different energetic states. It is also different compared to typical NMR relaxation theories, such as Redfield theory, since it explicitly studies the evolution of the bath oscillators, also at nonsecular frequencies. The simplicity of the method is a strength, considering also the fact that it allows investigation of longer correlation times, where the Redfield theory is not adaptable. The treatment of the complex coefficients associated with the Zeeman eigenstate vectors as classical *q*_*n*_/*p*_*n*_ variables and the conversion of the quantum mechanical matrix Hamiltonian into a ″classical″ Hamiltonian function, before arriving at the final expressions for motion of the density matrix elements, offers an alternative way of thinking about NMR spin dynamics. It also paves the way for continued work with path-integral-based methods where classical actions can be calculated for different trajectories of *q*_*n*_/*p*_*n*_.

## Supplementary Material



## Data Availability

Data and Software Availability: All source code and simulation data underlying the results and figures in the paper is available on 10.5281/zenodo.20155164.

## Notes

Details on the derivation of the equations of motion in the one- and two-spin case, as well as for the bath oscillators (PDF)
