# Supplementary material for: Semiclassical Molecular Dynamics for Condensed Phase NMR Spectroscopy
Source: J Chem Theory Comput. 2026 Sep 11;22(18):9302–10. doi: 10.1021/acs.jctc.6c01206 (PMC13602904; doi:10.1021/acs.jctc.6c01206)
Supplement: Supplementary file 1 [file ct6c01206_si_001.pdf]

SUPPORTING INFORMATION

Semiclassical Molecular Dynamics for condensed phase NMR spectroscopy

Hampus Karlsson*[1], Marco Cazzaniga[1], Michele Ceotto*[1]

[1]Dipartimento di Chimica, Università degli Studi di Milano, via Golgi 19, 20133 Milano, Italy

E-mail: michele.ceotto@unimi.it; carl.karlsson@unimi.it

Contents

S1 Derivation of equations of motion, one spin case **3**
S1.1 Details on the variable substitution 3
S1.2 Arriving at the final expression 4

S2 Equations of motion for the bath oscillators **6**

S3 Derivation of equations of motion, two-spin case **7**
S3.1 Conversion to classical Hamiltonian 7
S3.2 Two-spin density matrix 8

S4 Simulation times **9**

S1 Derivation of equations of motion, one spin case

S1.1 Details on the variable substitution

The variable substitution done in Eq. (10) in the main article aims to get rid of the H_B term as explained by [1]. Another consequence of the substitution is that the resulting integration variables correspond to the different elements of the single spin density matrix. We multiply the original equations of motion in Eq. (9a-d) in the main manuscript by their corresponding $2q_n$ or $2p_n$ variables to get $\frac{dq_n^2}{dt}$ and $\frac{dp_n^2}{dt}$.

$$\frac{dq_1^2}{dt} = 2q_1(H_Bp_1 - \tfrac{1}{2}H_{SB}p_1 + \tfrac{1}{2}\Omega p_1) = 2H_Bp_1q_1 - H_{SB}p_1q_1 + \Omega p_1q_1 \tag{S1.1}$$
$$\frac{dq_2^2}{dt} = 2q_2(H_Bp_2 + \tfrac{1}{2}H_{SB}p_2 - \tfrac{1}{2}\Omega p_2) = 2H_Bp_2q_2 + H_{SB}p_2q_2 - \Omega p_2q_2 \tag{S1.2}$$
$$\frac{dp_1^2}{dt} = 2p_1(-H_Bq_1 + \tfrac{1}{2}H_{SB}p_1 - \tfrac{1}{2}\Omega q_1) = -2H_Bp_1q_1 + H_{SB}p_1q_1 - \Omega p_1q_1 \tag{S1.3}$$
$$\frac{dp_2^2}{dt} = 2p_2(-H_Bq_2 - \tfrac{1}{2}H_{SB}q_2 + \tfrac{1}{2}\Omega q_2) = -2H_Bp_2q_2 - H_{SB}p_2q_2 + \Omega p_2q_2 \tag{S1.4}$$

Upon summation of Eq. (S1.1-4) the terms cancel as:

$$\frac{d}{dt}\left(q_1^2 + p_1^2 + q_2^2 + p_2^2\right) = 2H_Bp_1q_1 - H_{SB}p_1q_1 + \Omega p_1q_1 \tag{S1.5}$$
$$+ 2H_Bp_2q_2 + H_{SB}p_2q_2 - \Omega p_2q_2 \tag{S1.6}$$
$$-2H_Bp_1q_1 + H_{SB}p_1q_1 - \Omega p_1q_1 \tag{S1.7}$$
$$-2H_Bp_2q_2 - H_{SB}p_2q_2 + \Omega p_2q_2 = 0 \tag{S1.8}$$

The situation is the same for the difference term, $\left(q_1^2 + p_1^2 - q_2^2 - p_2^2\right)$, except for a sign switch of the blue terms. Now we get the equations of motion for the integration variables containing products and sums. For the motion of $(p_2q_1 - p_1q_2)$ the calculations becomes:

$$\frac{d}{dt}(p_2q_1 - p_1q_2) = \left(p_2\frac{dq_1}{dt} + q_1\frac{dp_2}{dt}\right) - \left(p_1\frac{dq_2}{dt} + q_2\frac{dp_2}{dt}\right) \tag{S1.9}$$
$$= \left[p_2(H_Bp_1 - \tfrac{1}{2}H_{SB}p_1 + \tfrac{1}{2}\Omega p_1) + q_1(-H_Bq_2 - \tfrac{1}{2}H_{SB}q_2 + \tfrac{1}{2}\Omega q_2)\right]$$
$$- \left[p_1(H_Bp_2 + \tfrac{1}{2}H_{SB}p_2 - \tfrac{1}{2}\Omega p_2) + q_2(-H_Bq_1 + \tfrac{1}{2}H_{SB}q_1 - \tfrac{1}{2}\Omega q_1)\right] \tag{S1.10}$$
$$= \left[\Omega p_1p_2 + \Omega q_1q_2 - H_{SB}p_1p_2 - H_{SB}q_1q_2\right] \tag{S1.11}$$
$$= \Omega(p_1p_2 + q_1q_2) - H_{SB}(p_1p_2 + q_1q_2) \tag{S1.12}$$

And similarly for $(p_1p_2 + q_1q_2)$ the calculations becomes:

$$\frac{d}{dt}(p_1p_2+q_1q_2) = \left(p_1\frac{dp_2}{dt}+p_2\frac{dp_1}{dt}\right)+\left(q_1\frac{dq_2}{dt}+q_2\frac{dq_1}{dt}\right) \tag{S1.13}$$

$$\begin{aligned}&= \left[p_1(-H_Bq_2-\tfrac{1}{2}H_{SB}q_2+\tfrac{1}{2}\Omega q_2)+p_2(-H_Bq_1+\tfrac{1}{2}H_{SB}q_1-\tfrac{1}{2}\Omega q_1)\right]\\ &- \left[q_1(H_Bp_2+\tfrac{1}{2}H_{SB}p_2-\tfrac{1}{2}\Omega p_2)+q_2(H_Bp_1-\tfrac{1}{2}H_{SB}p_1+\tfrac{1}{2}\Omega p_1)\right]\end{aligned} \tag{S1.14}$$

$$= [\Omega p_1q_2-\Omega p_2q_1-H_{SB}p_1q_2+H_{SB}p_2q_1] \tag{S1.15}$$

$$= -\Omega(p_2q_1-p_1q_2)+H_{SB}(p_2q_1-p_1q_2) \tag{S1.16}$$

S1.2 Arriving at the final expression

The two equations of interest for the $(p_1p_2+q_1q_2)$ and $(p_2q_1-p_1q_2)$ variables may be arranged as a 2×2 matrix system of equations. If we once again replace the variables again for readability, and set:

$$\tfrac{1}{2}(p_1p_2+q_1q_2) = S_{12} \tag{S1.17}$$

$$\tfrac{1}{2}(p_2q_1-p_1q_2) = D_{12} \tag{S1.18}$$

One may write the system as:

$$\frac{d}{dt}\begin{bmatrix}S_{12}\\D_{12}\end{bmatrix} = \begin{bmatrix}0 & H_{SB}-\Omega\\ \Omega-H_{SB} & 0\end{bmatrix}\begin{bmatrix}S_{12}\\D_{12}\end{bmatrix} \tag{S1.19}$$

The eigenvalues of the coefficient matrix become:

$$\lambda = \pm i(H_{SB}-\Omega) \tag{S1.20}$$

and its eigenvectors as:

$$\begin{bmatrix}i\\1\end{bmatrix}\ \mathit{and}\ \begin{bmatrix}-i\\1\end{bmatrix} \tag{S1.21}$$

The time-dependence of the S_{12} and D_{12} variables may be given by the solution:

$$\begin{bmatrix}S_{12}(t)\\D_{12}(t)\end{bmatrix} = C_1e^{\lambda_1 t}\begin{bmatrix}i\\1\end{bmatrix}+C_2e^{\lambda_2 t}\begin{bmatrix}-i\\1\end{bmatrix} \tag{S1.22}$$

where λ_1, λ_2 represent the eigenvalues, and we have used the eigenvectors from Eq. (S1.21). What is needed now are the coefficients C_1, C_2 which are obtained by solving:

$$Q\begin{bmatrix}C_1\\C_2\end{bmatrix} = \begin{bmatrix}i & -i\\1 & 1\end{bmatrix}\begin{bmatrix}C_1\\C_2\end{bmatrix} = \begin{bmatrix}S_{12}(0)\\D_{12}(0)\end{bmatrix} \tag{S1.23}$$

where Q represents the two eigenvectors arranged as a matrix. To solve Eq. (S1.23) one need to invert the matrix Q, and since it is a 2×2 matrix, we may use:

$$A = \begin{bmatrix} a & b \\ c & d \end{bmatrix}, \quad A^{-1} = \frac{1}{ad - bc} \begin{bmatrix} d & -b \\ -c & a \end{bmatrix}, \tag{S1.24}$$

Thus the inverse of the matrix Q is Q^{-1} and it is calculated as:

$$Q^{-1} = -\frac{i}{2} \begin{bmatrix} 1 & i \\ -1 & i \end{bmatrix} = \begin{bmatrix} -\frac{i}{2} & \frac{1}{2} \\ \frac{i}{2} & \frac{1}{2} \end{bmatrix} \tag{S1.25}$$

and C_1, C_2 become:

$$\begin{bmatrix} C_1 \\ C_2 \end{bmatrix} = \begin{bmatrix} -\frac{i}{2} & \frac{1}{2} \\ \frac{i}{2} & \frac{1}{2} \end{bmatrix} \begin{bmatrix} S_{12}(0) \\ D_{12}(0) \end{bmatrix} = \begin{bmatrix} \frac{D_{12}(0)}{2} - \frac{iS_{12}(0)}{2} \\ \frac{D_{12}(0)}{2} + \frac{iS_{12}(0)}{2} \end{bmatrix} \tag{S1.26}$$

The expressions for $S_{12}(t), D_{12}(t)$ may then be written:

$$S_{12}(t) = \left(\frac{D_{12}(0)}{2} - \frac{iS_{12}(0)}{2}\right) ie^{-i(H_{SB}-\Omega)t} - \left(\frac{D_{12}(0)}{2} + \frac{iS_{12}(0)}{2}\right) ie^{it(H_{SB}-\Omega)} \tag{S1.27}$$

$$D_{12}(t) = \left(\frac{D_{12}(0)}{2} - \frac{iS_{12}(0)}{2}\right) e^{-i(H_{SB}-\Omega)t} + \left(\frac{D_{12}(0)}{2} + \frac{iS_{12}(0)}{2}\right) e^{i(H_{SB}-\Omega)t} \tag{S1.28}$$

we recall that the S_{12} and D_{12} variables are just short hand notation for the composite integration variables, $(p_1p_2 + q_1q_2)/2$ and $(p_2q_1 - p_1q_2)/2$ and that they in turn correspond to the real and imaginary parts of the off-diagonal density matrix elements, as was explained in connection to Eq. (13) in the main manuscript. Thus, one may write the expression for the time-evolution of that density matrix element as:

$$\rho_{21}(t) = S_{12}(t) + iD_{12}(t) \tag{S1.29}$$

and if one substitute the terms on the RHS of Eq. (S1.29), one sees that the following terms cancel:

$$S_{12}(t) = \cancel{-\frac{iD_{12}(0)e^{iH_{SB}t}e^{-i\Omega t}}{2}} + \frac{iD_{12}(0)e^{-iH_{SB}t}e^{i\Omega t}}{2} \cancel{+\frac{S_{12}(0)e^{iH_{SB}t}e^{-i\Omega t}}{2}} + \frac{S_{12}(0)e^{-iH_{SB}t}e^{i\Omega t}}{2} \tag{S1.30}$$

$$iD_{12}(t) = \cancel{\frac{iD_{12}(0)e^{iH_{SB}t}e^{-i\Omega t}}{2}} + \frac{iD_{12}(0)e^{-iH_{SB}t}e^{i\Omega t}}{2} \cancel{-\frac{S_{12}(0)e^{iH_{SB}t}e^{-i\Omega t}}{2}} + \frac{S_{12}(0)e^{-iH_{SB}t}e^{i\Omega t}}{2} \tag{S1.31}$$

and that the remaining terms can be summed up as:

$$\rho_{21}(t) = (S_{12}(0) + iD_{12}(0))e^{i(\Omega - H_{SB})t} \tag{S1.32}$$

which may also be written as:

$$\rho_{21}(t) = \rho_{21}(0)e^{i(\Omega - H_{SB})t} \tag{S1.33}$$

This is the expression that has been used in the simulations for the motion of the lower diagonal matrix element in the single spin case.

S2 Equations of motion for the bath oscillators

The matrix Hamiltonian in Eq. (4) in the main manuscript was defined as:

$$\hat{H} = \begin{bmatrix} H_B + \frac{\Omega}{2} - \frac{H_{SB}}{2} & 0 \\ 0 & H_B - \frac{\Omega}{2} + \frac{H_{SB}}{2} \end{bmatrix} \tag{S2.1}$$

If we instead of the H_B and H_{SB} symbols write out the sums for the bath oscillators explicitly it becomes:

$$\hat{H} = \begin{bmatrix} \sum_j \frac{1}{2}(P_j^2 + \omega_j^2 Q_j^2) + \frac{\Omega}{2} - \frac{1}{2}\sum_j C_j Q_j & 0 \\ 0 & \sum_j \frac{1}{2}(P_j^2 + \omega_j^2 Q_j^2) - \frac{\Omega}{2} + \frac{1}{2}\sum_j C_j Q_j \end{bmatrix} \tag{S2.2}$$

and if we disregard the chemical shift term, Ω from the spin system part of the Hamiltonian and only consider the bath and system-bath terms for the jth oscillator, this Hamiltonian simplifies to:

$$\hat{H}_{Bath} = \begin{bmatrix} \frac{1}{2}(P_j^2 + \omega_j^2 Q_j^2) - \frac{1}{2}C_j Q_j & 0 \\ 0 & \frac{1}{2}(P_j^2 + \omega_j^2 Q_j^2) + \frac{1}{2}C_j Q_j \end{bmatrix} \tag{S2.3}$$

When this "classical" Hamiltonian function for this matrix Hamiltonian is calculated following Eq.s (6-8) in the main manuscript we get:

$$\begin{aligned} H_{CLBath} = &-\tfrac{1}{4}C_jQ_jp_1^2 + \tfrac{1}{4}C_jQ_jp_2^2 - \tfrac{1}{4}C_jQ_jq_1^2 + \tfrac{1}{4}C_jQ_jq_2^2 + \tfrac{1}{4}P_j^2p_1^2 + \tfrac{1}{4}P_j^2p_2^2 \\ &+ \tfrac{1}{4}P_j^2q_1^2 + \tfrac{1}{4}P_j^2q_2^2 + \tfrac{1}{4}Q_j^2\omega_j^2p_1^2 + \tfrac{1}{4}Q_j^2\omega_j^2p_2^2 + \tfrac{1}{4}Q_j^2\omega_j^2q_1^2 + \tfrac{1}{4}Q_j^2\omega_j^2q_2^2 \end{aligned} \tag{S2.4}$$

The equations of motion are calculated according to:

$$\dot{Q}_n = \frac{\partial H_{CLBath}}{\partial P_n} \quad \dot{P}_n = -\frac{\partial H_{CLBath}}{\partial Q_n} \tag{S2.5}$$

and one gets:

$$\dot{Q}_j = \frac{P_j}{2}(q_1^2 + q_2^2 + p_1^2 + p_2^2) \tag{S2.6}$$

$$\dot{P}_j = -\frac{Q_j}{2}\omega^2(q_1^2 + q_2^2 + p_1^2 + p_2^2) + \frac{C_j}{4}(q_1^2 + p_1^2 - q_2^2 - p_2^2) \tag{S2.7}$$

As it was explained in the main manuscript the $(q_1^2 + q_2^2 + p_1^2 + p_2^2)$ term representing the sum of the diagonal terms of the density matrix will remain constant and never change, whereas the $(q_1^2 + p_1^2 - q_2^2 - p_2^2)$ will evaluate to zero due to the absence of diagonal terms in the original matrix Hamiltonian. This means that Eq. (S2.6 & 7) can be written as the 2×2 system:

$$\frac{d}{dt}\begin{bmatrix} Q_j \\ P_j \end{bmatrix} = \begin{bmatrix} 0 & 1 \\ -\omega^2 & 0 \end{bmatrix}\begin{bmatrix} Q_j \\ P_j \end{bmatrix} \tag{S2.8}$$

for which we have used the solution presented in Eq. (15) in the main manuscript.

S3 Derivation of equations of motion, two-spin case

S3.1 Conversion to classical Hamiltonian

In full analogy with the one-spin case we may also derive equations of motion for the density matrix elements associated with the -1 quantum coherence for the spins in a coupled spin pair. This, is obtained via conversion to a "classical" Hamiltonian and variable substitution, just as in the one-spin case. The two-spin Hamiltonian considered is of the type:

$$\hat{H} = \Omega_1 \hat{I}_{1z} + \Omega_2 \hat{I}_{2z} + \pi J 2 \hat{I}_{1z} \hat{I}_{2z} \tag{S3.1}$$

Its matrix representation is:

$$\hat{H} = \frac{1}{2} \begin{bmatrix} J\pi + \Omega_1 + \Omega_2 & 0 & 0 & 0 \\ 0 & -J\pi + \Omega_1 - \Omega_2 & 0 & 0 \\ 0 & 0 & -J\pi - \Omega_1 + \Omega_2 & 0 \\ 0 & 0 & 0 & J\pi - \Omega_1 - \Omega_2 \end{bmatrix} \tag{S3.2}$$

And if a bath term, H_B is added to the diagonal, and spin-bath terms H_{SB1}, H_{SB2} are allowed to couple to the $\hat{I}_{1z}$ and $\hat{I}_{2z}$ operators we get the two spin Hamiltonian that corresponds to the one-spin Hamiltonian in Eq. (4) in the main manuscript:

$$\hat{H} = \begin{bmatrix} H_B - \frac{1}{2}(H_{SB1} + H_{SB2} - J\pi - \Omega_1 - \Omega_2) & 0 & 0 & 0 \\ 0 & H_B - \frac{1}{2}(H_{SB1} - H_{SB2} + J\pi - \Omega_1 + \Omega_2) & 0 & 0 \\ 0 & 0 & H_B + \frac{1}{2}(H_{SB1} - H_{SB2} - J\pi - \Omega_1 + \Omega_2) & 0 \\ 0 & 0 & 0 & H_B + \frac{1}{2}(H_{SB1} + H_{SB2} + J\pi - \Omega_1 - \Omega_2) \end{bmatrix} \tag{S3.3}$$

And if we in analogy with the one-spin case, also here write the Zeemann eigenstate in terms of q_n and p_n, here extended for two spins:

$$|\Psi\rangle = \frac{1}{2} \begin{bmatrix} q_1 + ip_1 \\ q_2 + ip_2 \\ q_3 + ip_3 \\ q_4 + ip_4 \end{bmatrix} \tag{S3.4}$$

And then use the formula for the expectation value:

$$H_{CL} = \langle \Psi | \hat{H} | \Psi \rangle \tag{S3.5}$$

To obtain the "classical" Hamiltonian function in the two-spin case, we get:

$$
\begin{aligned}
H_{CL} = {} & \tfrac{1}{2}H_B p_1^2 + \tfrac{1}{2}H_B p_2^2 + \tfrac{1}{2}H_B p_3^2 + \tfrac{1}{2}H_B p_4^2 + \tfrac{1}{2}H_B q_1^2 + \tfrac{1}{2}H_B q_2^2 + \tfrac{1}{2}H_B q_3^2 + \tfrac{1}{2}H_B q_4^2 \\
& - \tfrac{1}{4}H_{SB1} p_1^2 - \tfrac{1}{4}H_{SB1} p_2^2 + \tfrac{1}{4}H_{SB1} p_3^2 + \tfrac{1}{4}H_{SB1} p_4^2 - \tfrac{1}{4}H_{SB1} q_1^2 - \tfrac{1}{4}H_{SB1} q_2^2 + \tfrac{1}{4}H_{SB1} q_3^2 + \tfrac{1}{4}H_{SB1} q_4^2 \\
& - \tfrac{1}{4}H_{SB2} p_1^2 + \tfrac{1}{4}H_{SB2} p_2^2 - \tfrac{1}{4}H_{SB2} p_3^2 + \tfrac{1}{4}H_{SB2} p_4^2 - \tfrac{1}{4}H_{SB2} q_1^2 + \tfrac{1}{4}H_{SB2} q_2^2 - \tfrac{1}{4}H_{SB2} q_3^2 + \tfrac{1}{4}H_{SB2} q_4^2 \\
& + \tfrac{1}{4}J\pi p_1^2 - \tfrac{1}{4}J\pi p_2^2 - \tfrac{1}{4}J\pi p_3^2 + \tfrac{1}{4}J\pi p_4^2 + \tfrac{1}{4}J\pi q_1^2 - \tfrac{1}{4}J\pi q_2^2 - \tfrac{1}{4}J\pi q_3^2 + \tfrac{1}{4}J\pi q_4^2 \\
& + \tfrac{1}{4}\Omega_1 p_1^2 + \tfrac{1}{4}\Omega_1 p_2^2 - \tfrac{1}{4}\Omega_1 p_3^2 - \tfrac{1}{4}\Omega_1 p_4^2 + \tfrac{1}{4}\Omega_1 q_1^2 + \tfrac{1}{4}\Omega_1 q_2^2 - \tfrac{1}{4}\Omega_1 q_3^2 - \tfrac{1}{4}\Omega_1 q_4^2 \\
& + \tfrac{1}{4}\Omega_2 p_1^2 - \tfrac{1}{4}\Omega_2 p_2^2 + \tfrac{1}{4}\Omega_2 p_3^2 - \tfrac{1}{4}\Omega_2 p_4^2 + \tfrac{1}{4}\Omega_2 q_1^2 - \tfrac{1}{4}\Omega_2 q_2^2 + \tfrac{1}{4}\Omega_2 q_3^2 - \tfrac{1}{4}\Omega_2 q_4^2
\end{aligned} \tag{S3.6}
$$

And the associated equations of motion become:

$$\dot{q}_1 = H_B p_1 - \tfrac{1}{2}H_{SB1}p_1 - \tfrac{1}{2}H_{SB2}p_1 + \tfrac{1}{2}Jp_1\pi + \tfrac{1}{2}\Omega_1 p_1 + \tfrac{1}{2}\Omega_2 p_1 \tag{S3.7}$$

$$\dot{q}_2 = H_B p_2 - \tfrac{1}{2}H_{SB1}p_2 + \tfrac{1}{2}H_{SB2}p_2 - \tfrac{1}{2}Jp_2\pi + \tfrac{1}{2}\Omega_1 p_2 - \tfrac{1}{2}\Omega_2 p_2 \tag{S3.8}$$

$$\dot{q}_3 = H_B p_3 + \tfrac{1}{2}H_{SB1}p_3 - \tfrac{1}{2}H_{SB2}p_3 - \tfrac{1}{2}Jp_3\pi - \tfrac{1}{2}\Omega_1 p_3 + \tfrac{1}{2}\Omega_2 p_3 \tag{S3.9}$$

$$\dot{q}_4 = H_B p_4 + \tfrac{1}{2}H_{SB1}p_4 + \tfrac{1}{2}H_{SB2}p_4 + \tfrac{1}{2}Jp_4\pi - \tfrac{1}{2}\Omega_1 p_4 - \tfrac{1}{2}\Omega_2 p_4 \tag{S3.10}$$

$$\dot{p}_1 = -H_B q_1 + \tfrac{1}{2}H_{SB1}q_1 + \tfrac{1}{2}H_{SB2}q_1 - \tfrac{1}{2}J\pi q_1 - \tfrac{1}{2}\Omega_1 q_1 - \tfrac{1}{2}\Omega_2 q_1 \tag{S3.11}$$

$$\dot{p}_2 = -H_B q_2 + \tfrac{1}{2}H_{SB1}q_2 - \tfrac{1}{2}H_{SB2}q_2 + \tfrac{1}{2}J\pi q_2 - \tfrac{1}{2}\Omega_1 q_2 + \tfrac{1}{2}\Omega_2 q_2 \tag{S3.12}$$

$$\dot{p}_3 = -H_B q_3 - \tfrac{1}{2}H_{SB1}q_3 + \tfrac{1}{2}H_{SB2}q_3 + \tfrac{1}{2}J\pi q_3 + \tfrac{1}{2}\Omega_1 q_3 - \tfrac{1}{2}\Omega_2 q_3 \tag{S3.13}$$

$$\dot{p}_4 = -H_B q_4 - \tfrac{1}{2}H_{SB1}q_4 - \tfrac{1}{2}H_{SB2}q_4 - \tfrac{1}{2}J\pi q_4 + \tfrac{1}{2}\Omega_1 q_4 + \tfrac{1}{2}\Omega_2 q_4 \tag{S3.14}$$

S3.2 Two-spin density matrix

In the two-spin case it is these density matrix elements, associated with the -1 quantum coherence of the first spin that we are interested in order to simulate the split peak from spin one.

$$
\hat{H} = \frac{1}{4}\begin{bmatrix}
p_1^2 + q_1^2 & p_1p_2 + ip_1q_2 - ip_2q_1 + q_1q_2 & p_1p_3 + ip_1q_3 - ip_3q_1 + q_1q_3 & p_1p_4 + ip_1q_4 - ip_4q_1 + q_1q_4 \\
p_1p_2 - ip_1q_2 + ip_2q_1 + q_1q_2 & p_2^2 + q_2^2 & p_2p_3 + ip_2q_3 - ip_3q_2 + q_2q_3 & p_2p_4 + ip_2q_4 - ip_4q_2 + q_2q_4 \\
\boxed{p_1p_3 - ip_1q_3 + ip_3q_1 + q_1q_3} & p_2p_3 - ip_2q_3 + ip_3q_2 + q_2q_3 & p_3^2 + q_3^2 & p_3p_4 + ip_3q_4 - ip_4q_3 + q_3q_4 \\
p_1p_4 - ip_1q_4 + ip_4q_1 + q_1q_4 & \boxed{p_2p_4 - ip_2q_4 + ip_4q_2 + q_2q_4} & p_3p_4 - ip_3q_4 + ip_4q_3 + q_3q_4 & p_4^2 + q_4^2
\end{bmatrix} \tag{S3.15}
$$

It is thus motion of the terms, $(q_1q_3 + p_1p_3)$, $(p_3q_1 - p_1q_3)$, $(q_2q_4 + p_2p_4)$ and $(p_4q_2 - p_2q_4)$ that we are interested in. In full analogy with the one-spin case, the equations of motion these matrix elements become:

$$\frac{d}{dt}(q_1q_3 + p_1p_3) = H_{SB1}(p_3q_1 - p_1q_3) - J\pi(p_3q_1 - p_1q_3) - \Omega_1(p_3q_1 - p_1q_3) \tag{S3.16}$$

$$\frac{d}{dt}(p_3q_1 - p_1q_3) = -H_{SB1}(q_1q_3 + p_1p_3) + J\pi(q_1q_3 + p_1p_3) + \Omega_1(q_1q_3 + p_1p_3) \tag{S3.17}$$

$$\frac{d}{dt}(q_2q_4 + p_2p_4) = H_{SB1}(p_4q_2 - p_2q_4) + J\pi(p_4q_2 - p_2q_4) - \Omega_1(p_4q_2 - p_2q_4) \tag{S3.18}$$

$$\frac{d}{dt}(p_4q_2 - p_2q_4) = -H_{SB1}(q_2q_4 + p_2p_4) - J\pi(q_2q_4 + p_2p_4) + \Omega_1(q_2q_4 + p_2p_4) \tag{S3.19}$$

And just as in the one-spin case if one renames the variables as:

$$\tfrac{1}{4}(q_1q_3 + p_1p_3) = S_{13} \tag{S3.20}$$
$$\tfrac{1}{4}(p_3q_1 - p_1q_3) = D_{13} \tag{S3.21}$$
$$\tfrac{1}{4}(q_2q_4 + p_2p_4) = S_{24} \tag{S3.22}$$
$$\tfrac{1}{4}(p_4q_2 - p_2q_4) = D_{24} \tag{S3.23}$$

Equations (S2.16-19) can be rewritten as matrix equations:

$$\frac{d}{dt}\begin{bmatrix} S_{13} \\ D_{13} \end{bmatrix} = \begin{bmatrix} 0 & H_{SB1} - J\pi - \Omega_1 \\ -H_{SB1} + J\pi + \Omega_1 & 0 \end{bmatrix}\begin{bmatrix} S_{13} \\ D_{13} \end{bmatrix} \tag{S3.24}$$

$$\frac{d}{dt}\begin{bmatrix} S_{24} \\ D_{24} \end{bmatrix} = \begin{bmatrix} 0 & H_{SB1} + J\pi - \Omega_1 \\ -H_{SB1} - J\pi + \Omega_1 & \end{bmatrix}\begin{bmatrix} S_{24} \\ D_{24} \end{bmatrix} \tag{S3.25}$$

With solutions:

$$S_{13}(t) = \left(\frac{S_{13}(0)}{2} + \frac{iD_{13}(0)}{2}\right) e^{-i(H_{SB1}-J\pi-\Omega_1)t} + \left(\frac{S_{13}(0)}{2} - \frac{iD_{13}(0)}{2}\right) e^{i(H_{SB1}-J\pi-\Omega_1)t} \tag{S3.26}$$
$$D_{13}(t) = \left(\frac{D_{13}(0)}{2} - \frac{iS_{13}(0)}{2}\right) e^{-i(H_{SB1}-J\pi-\Omega_1)t} + \left(\frac{D_{13}(0)}{2} + \frac{iS_{13}(0)}{2}\right) e^{i(H_{SB1}-J\pi-\Omega_1)t} \tag{S3.27}$$

And

$$S_{24}(t) = \left(\frac{S_{24}(0)}{2} + \frac{iD_{24}(0)}{2}\right) e^{-i(H_{SB1}+J\pi-\Omega_1)t} + \left(\frac{S_{24}(0)}{2} - \frac{iD_{24}(0)}{2}\right) e^{i(H_{SB1}+J\pi-\Omega_1)t} \tag{S3.28}$$
$$D_{24}(t) = \left(\frac{D_{24}(0)}{2} - \frac{iS_{24}(0)}{2}\right) e^{-t(H_{SB1}+J\pi-\Omega_1)t} + \left(\frac{D_{24}(t)}{2} + \frac{iS_{24}(0)}{2}\right) e^{i(H_{SB1}+J\pi-\Omega_1)t} \tag{S3.29}$$

Obtained in the same manner as in Eq. (S1.22-S1.26). If the D_{13} and D_{24} terms are multiplied by i as to represent the imaginary part in the density matrix, one may arrive at the final expression for simulating the FID signal for the first coupled spin in the spin pair.

$$S(t) = \rho_{31}(0)e^{i(\Omega_1 - H_{SB1} + J\pi)t} + \rho_{42}(0)e^{i(\Omega_1 - H_{SB1} - J\pi)t} \tag{S3.30}$$

Where it is easy to see that $J\pi$ will create two distinct peaks in the spectrum.

S4 Simulation times

The simulated spectra presented in section 4.2 and 4.3 of the main manuscript were simulated using multi-threaded C++ code or as hybrid CUDA/C++ code on different machines as described in section 3 of the main manuscript (see the Zenodo link in the "Data and Software Availability" section of the main manuscript for access to the source code used). Simulation times and settings for the different simulations are outlined in table S4.1 below:

Table S4.1: Overview of simulation times

correlation time (ns)	number of spins	simulated time (ms)	number of trajectories	oscillators in bath	program / application	computation time (hours)
100	one	10.5	2048	4096	CUDA/C++	9.1
50	one	10.5	2048	4096	CUDA/C++	8.8
10	one	21	2048	4096	CUDA/C++	17.8
10	two	42	1024	8192	C++	48

Comment about scalability and performance on different machines: Preliminary tests with hybrid OpenMP/OpenMPI C++ applications have been done where different trajectories have been distributed both between different threads and between different nodes in a computer cluster. These tests indicate a linear decrease in computation time with inclusion of more cores/nodes. Yet, these test are of highly preliminary nature and tests with many nodes have not been performed. Highly parallel, hybrid OpenMP/OpenMPI C++ code would be necessary for potential future work with numerical propagation of anharmonic baths and more complicated spin systems. Regarding usage of CUDA cores and GPU resources it is likely that this is most advantageous in the unique situation of transverse magnetization and no change in spin state studied, as in this work. Usage of GPU/CUDA is probably less beneficial in more complicated scenarios such as change in spin state, where information about the spin state would need to be copied between CPU/GPU memory at each time-step.

References

1. H. Wang, X. Song, D. Chandler, and W. H. Miller, "Semiclassical study of electronically nonadiabatic dynamics in the condensed-phase: Spin-boson problem with debye spectral density," The Journal of Chemical Physics 110, 4828–4840 (1999).
